# Comparison of two suicide screening instruments for identifying high-risk individuals in prison

**DOI:** 10.3389/fpsyt.2024.1362928

**Published:** 2024-06-27

**Authors:** Joscha Hausam, Daniela Calvano, Annette Opitz-Welke

**Affiliations:** ^1^ Institute of Forensic Psychiatry, Charité - Universitätsmedizin Berlin, Berlin, Germany; ^2^ Department of Psychiatry and Psychotherapy, Prison Hospital Berlin, Berlin, Germany

**Keywords:** suicide, suicide prevention, prison, VISCI, SIRAS, suicide screening, offender

## Abstract

Increased suicide rates in prison are a serious concern. Early identification of inmates at risk is a component of effective suicide prevention. The present study examined two suicide screening instruments in a sample of men in the Berlin, Germany, prison system (n = 289). The Screening for Initial Risk Assessment (SIRAS) identified significantly more high-risk inmates than the Vienna Instrument for Suicidality in Correctional Institutions (VISCI) (66 vs. 24). The results further show that the agreement in the classification was evident only in inmates with suicidal ideation, but was otherwise quite low. This can be explained by the fact that the instruments differ in terms of the risk factors taken into account. Finally, it was found that inmates classified as high risk received more monitoring and psychiatric or psychological support, which supports the construct validity of the instruments. As there were no deaths by suicide in the sample, no statistical information on the predictive validity of the instruments could be provided. Although research in this area is challenging, methodologically sound studies are needed to inform practice.

## 1 Introduction

Suicide is the leading cause of death in prisons worldwide, and prisoners are at much higher risk of death by suicide than the general population (1–4). Given the high rate in prisons, it is important to understand the possible causes and risk factors in order to prevent death by suicide. According to the crisis and illness model (5), suicidality can be understood as the endpoint of two different developments: the crisis model assumes a previously healthy personality that becomes suicidal in the context of stressful life events. Particularly in pre-trial detention or at the beginning of imprisonment, various stress factors (e.g., the high degree of heteronomy or the lack of contact with people outside the prison) can lead to a personal, seemingly unsolvable crisis. The illness model locates the cause in the psychopathology of a mental disorder. Significantly increased prevalence of psychiatric illnesses and substance use disorders among people in prison is well documented (6, 7). Accordingly, Favril (4) concluded that prison-specific stressors may exacerbate suicide risk in an already vulnerable population characterized by complex health and social care needs.

Studies of individual risk factors for death by suicide in prison are consistent with these etiopathogenetic assumptions. A recent meta-analysis of 77 studies, including 27 countries and 35,351 deaths by suicide, identified a psychiatric diagnosis, substance use disorder, and pretrial status as strongest risk factors (8). In addition, suicidal ideation while incarcerated, previous suicide attempts or non-suicidal self-injury, and solitary confinement were identified as significant risk factors. These findings are largely consistent with and extend previous reviews (7, 9, 10). Thus, current models of suicidal behavior in inmates incorporate personal vulnerabilities, clinical factors, criminological and institutional factors, and emphasize their interactions (11).

Effective suicide prevention in correctional settings requires the early identification of high-risk individuals and the provision of appropriate suicide prevention interventions (12). Given the limited number of professionals and resources in prisons, screening instruments have been proposed as one measure to better identify “at risk” individuals (13, 14). While proponents see this as an easy measure to implement, critics argue that suicide screening may be of little use because it is costly and relies on the belief that suicide risk can be identified (15). In fact, research over the past 50 years has shown that valid prediction of suicidal ideation and behavior based on risk factors is only slightly better than chance (16). This appears to be particularly problematic when correctional facilities use screening instruments that have not been validated (17, 18).

Gould et al. (19) examined the predictive validity of suicide screening instruments in correctional facilities, updating an earlier review (20). Both reviews point out that there are few instruments and a lack of methodologically sound studies. Specifically, the low prevalence of deaths by suicide and the high prevalence of risk factors among prisoners complicate the testing of instruments. As a result, no meta-analytical evaluation was carried out in the reviews. Nonetheless, Gould et al. (19) identified eight different instruments and concluded that two instruments can be recommended. These are the *Vienna Instrument for Suicidality in Correctional Institutions* (VISCI; 21, 22) and the Dutch Suicide Screening (23) or its modified version *Screening for Initial Risk Assessment* (SIRAS; 24). This recommendation was recently confirmed by Riblet et al. (25) using likelihood ratio analysis. However, there are few independent studies on the effectiveness of these tools as a preventive measure. An exception is the study by DezsöCheck that all equations and special characters are displayed correctly. et al. (26), in which the use of SIRAS led to a more targeted and efficient allocation of interventions (e.g., psychological counseling) in the Berlin prison system. At the same time, the application was perceived as a burden by staff, highlighting the importance of internal training.

In methodological terms, suicide screening is based on the classification of individuals using a 2x2 cross-tabulation to identify those who are truly at risk (i.e. the sensitivity of the instrument) and those who are not (i.e. the specificity). The determination of an optimal cut-off is always a trade-off between these two parameters and is accompanied by an inevitable misclassification. In practice, a misclassification can have considerable consequences (20). To the best of our knowledge, there are no studies comparing the classification of suicide screening instruments. Therefore, the present study compares the VISCI and SIRAS in a male prison sample. The primary objective is to examine the extent to which the instruments identify the same individuals as high risk (i.e., concordance of classification). In addition, associations with relevant measures in the prison system that are related to death by suicide and suicide prevention are examined.

## 2 Methods

### 2.1 Sample

The sample consists of 289 men who were admitted to Moabit Prison in Berlin, Germany, between 1 October 2015 and 31 March 2016. This was a full survey. Moabit is primarily a remand prison for adult men (n = 177, 61.2%), but it also houses male prisoners at the beginning of their sentences (n = 112, 38.8%). The first group has not yet been convicted but has been arrested because there is a strong suspicion of a crime and a reason for arrest (e.g., risk of flight or concealment). The second group consists of men held on unexecuted warrants for legally binding prison sentences. Although there are differences in legal status, the inmates in both groups are initially treated similarly: They are usually locked up for 23 hours, placed in solitary confinement, and have limited contact with others (less so for those with convictions). All inmates are examined by a prison physician and a social worker immediately upon admission (the same day or the next workday at the latest). This intake assessment includes a physical and mental health check and a social background review. Special measures may be ordered in the event of abnormalities (e.g., withdrawal symptoms in the case of addiction problems, psychological abnormalities such as panic or suicidal thoughts). These may include regular day or night observation by prison officers (e.g., the prisoner is checked in his cell every 2 hours), prescription of medication (e.g., antidepressant for psychological stress), consultation with a psychiatrist or psychologist for further clarification, or referral to the prison psychiatry for acute psychiatric problems. It is important to note that no suicide screening tool was routinely used at the facility at that time, but clinical assessment of suicidality is regularly performed (e.g., suicidal ideation is asked).

Data for this study were collected by a physician independent of the Moabit Prison who visited the facility once a week during the six-month study period and reviewed the records of all newly admitted prisoners (i.e., within approximately 1 week). Two suicide screening instruments were administered retrospectively based on the medical and personal records. The study physician had no personal contact with the prisoners, i.e. the assessments are based mostly on the documented intake examinations. Other events that occurred between admission and data collection and were noted in the file were also included (e.g., suicidal acts). This was a one-time assessment. A re-identification of the cases was not possible. The order of data collection corresponds to the materials described below. For data protection reasons and to ensure anonymity, no other sample characteristics such as age, nationality, etc. was collected.

### 2.2 Materials

The Scale for Initial Risk Assessment for Suicide (SIRAS; 24) is a modified version of the Dutch Suicide Screening (23) and consists of six items (presence rating): age 40 years or older, no fixed address or residence prior to incarceration, no or one prior incarceration, history of hard drug abuse in combination with soft drugs and/or alcohol, previous suicide attempts or non-suicidal self-injury, and current suicide attempt or suicidal ideation. The items are summed to produce a risk score (1 point per item, current suicide attempts or ideation 3 points). The SIRAS score ranges from 0 to 8. A score of 3 points or more indicates high risk, which in practice should result in immediate referral to a specialized psychological or psychiatric service. A predictive accuracy of AUC = .88 was reported in the validation study in a pretrial sample (n = 60, 30 deaths by suicide) at Moabit Prison in Berlin, Germany (24).

The Viennese Instrument for Suicidality in Correctional Institutions (VISCI; 21, 22) was developed in and for the Austrian correctional system. This study used an unpublished version of the VISCI questionnaire and scoring sheet. Due to ambiguities in the application, we contacted the first author of the VISCI, who provided us with this version. There are two major changes (or clarifications) from the published version: Suicidal ideation (“Are you currently thinking about taking your own life?”) and prison status (pretrial/sentenced) were included in the calculation of the total score. Although these items were also asked in the original questionnaire, they were not given a weighted score in the final model (at least this was not clear to us in the publication). Of the 22 VISCI questions, 13 are rated in the screening assessment: pretrial or sentenced custody status, any previous violent offense, violent index offense, drug index offense, property index offense, previous incarcerations, marital status, employed before incarceration, contact with psychiatrist or psychiatry, suicide attempt, suicide threat, psychiatric diagnosis, and current suicidal ideation. Items are weighted (depending on custody status) and summed to produce a risk score. Custody status (pretrial/sentenced) was weighted with a score of -1.940 and -1.890 and suicidal ideation was weighted with 7.225 (pretrial) and 6.460 (sentenced). Generally, the items can have a negative or positive sign, i.e. they reduce or increase the risk of suicide. The weights of the other items can be found in the published work (21, 22). The VISCI score ranges from -3.72 to 17.32 (pretrial) and -2.96 to 16.68 (sentenced). The assessment follows a traffic light system: Values below 1.5 indicate no suicide risk (green light), values greater than or equal to 1.5 indicate a low suicide risk (yellow light), values of 3.5 or greater indicate a high suicide risk (red light). In practice, if the traffic light is yellow, it is recommended that the inmate is not placed in a single cell and goes to work during the day, and if the traffic light is red, that the inmate is immediately referred to a specialist service (psychiatrist, psychologist or social worker). In the developmental study (n = 660 with 220 deaths by suicide), the predictive accuracy was AUC = .88 in the pretrial sample and AUC = .89 in the sentenced sample (21). In a validation study (n = 165 with 55 deaths by suicide), the values were slightly lower (AUC =.76 and,.84 respectively; 22).

Conceptually, it is important to distinguish between suicide risk and acute suicidality (5). Suicide risk (sometimes also called basic suicidality) refers to those who are generally at risk for suicidal thoughts or behaviors in the future. This is derived from membership in a risk group or the presence of risk factors. Screening instruments are largely aimed at determining the suicide risk. Acute suicidality is defined as a current threat (e.g., due to a psychopathological condition) that manifests itself in suicidal ideations or actions. Acute suicidality could be further differentiated (e.g., does the person talk about it, can it be inferred from their behavior, were there indications from others), but this is not represented in the instruments (but should of course be part of the clinical assessment). In both instruments, acute suicidality is assessed with one item each, which, as mentioned above, requires immediate action (i.e., the cut-off is exceeded with this item in both instruments). However, there is a significant difference in the operationalization of the two instruments: While the VISCI asks only for suicidal ideation, the SIRAS explicitly includes suicidal ideation and behavior (i.e., current suicide attempts). Due to the retrospective and file-based approach of this study and the associated difficulty of differentiation, suicide attempts were also included in the VISCI rating. This is based on the assumption that suicidality represents a spectrum, i.e. there are usually thoughts before actions.

To further test the validity of the suicide screening instruments, additional variables were collected that may serve as surrogate markers for suicidality. Surrogate markers are a measure that may correlate with a real clinical endpoint but does not necessarily have a guaranteed relationship. In principle, this procedure is based on two assumptions: First, despite the increased prevalence compared to the general population, death by suicide is still a rare event also in the prison system, so it was not expected that there would be a death by suicide in the sample to examine predictive validity. Secondly, this allowed us to check the “congruence” between the clinical and structured assessment of suicide risk as indicated by the ordered measures. The following measures were recorded as indicators of suicide risk (i.e., surrogate markers): observation, night-time checks^1^, consultation with a psychiatrist or psychologist, prescription of medication, and admission to the prison psychiatric ward.

### 2.3 Statistical analysis

Statistical analyses were performed using SPSS for Windows (version 29). There was no missing data. In addition to descriptive analyses of the distribution of scores, frequencies were calculated and reported. For the surrogate markers, additional chi-squared tests were performed to determine whether the measures were significantly more frequently ordered in the risk groups. Chi-squared analyses were performed independently for each surrogate marker and for SIRAS and VISCI respectively. For example, it was examined whether night watch was ordered more often for inmates classified as high risk by SIRAS than for low-risk inmates. Due to the small group size of the high-risk groups (especially for VISCI), the risk estimates of the instruments were not compared with respect to the surrogate markers. The alpha level was set at.05.

## 3 Results

Descriptive statistics and risk levels of the VISCI and SIRAS are shown in **Table 1**. Please note that negative scores are possible on the VISCI due to the weighting of the items. In fact, all inmates “start” with a negative score due to the negative custody status item. Most of the sample was classified as low risk by the VISCI (n = 253, 87.5%) and the SIRAS (n = 223, 77.2%). The VISCI identified 24 inmates (8.3%) of the sample as high risk (red light) and 12 inmates (4.2%) as moderate risk (yellow light), while the SIRAS identified 66 (22.8%) as high risk. As described above, suicidal ideation and behavior is included in both instruments and is weighted so heavily that an immediate high-risk or red light classification follows if the answer is yes. This was the case in 20 inmates. Therefore, of the 24 red light (VISCI) inmates, suicidal ideation or behavior was not confirmed in four inmates. For all four of these inmates, the combination of a previous suicide attempt and a psychiatric diagnosis led to the classification as high risk. In almost all yellow light inmates (92%), a psychiatric diagnosis was affirmed, otherwise there was no clear pattern in the distribution of risk factors.

**Table 1:** Risk Scores and Classification of the VISCI and SIRAS.

	M	SD	Min-Max	Low (green)	Risk	High (red)
					(yellow)	
VISCI	-0.31	2.91	-3.72 – 14.05	253 (87.5%)	12 (4.2%)	24 (8.3%)
SIRAS	1.90	1.28	0 - 7	223 (77.2%)	–	66 (22.8%)

VISCI, Viennese Instrument for Suicidality in Correctional Institutions; SIRAS, Scale for Initial Risk Assessment for Suicide. There are three risk categories in VISCI (green, low, red) and two in SIRAS (low, high).

M, mean; SD, standard deviation.

In the SIRAS, the suicidality item was less of a “decisive factor” for a high risk classification: of the 66 high-risk patients, 46 were inmates with no documented suicidal ideation or attempt and scored 3 or 4 points on a combination of the other items. The following item frequencies were found for high-risk inmates: no or one previous incarceration (75.8%), no fixed address or residence (74.2%), age 40 or older (50.0%), history of hard drug abuse in combination with soft drugs and/or alcohol (48.5%), and previous suicide attempt (37.9%).

The results of the classification concordance analyses are shown in **Table 2**. For the convenience of the reader, only the column percentages are shown here. The full table can be found in the electronic supplementary material (ESM 1). It should first be noted that 23 inmates were consistently identified as high risk by both instruments. Only one inmate was identified as high risk (red) and eight inmates were identified as moderate risk (yellow) by the VISCI but not by the SIRAS. From the VISCI perspective, concordance is high for high-risk inmates, while medium-risk inmates in the VISCI are twice as likely to be missed by SIRAS as high-risk individuals. Conversely, 39 green and four yellow inmates were identified as high risk by the SIRAS. Taken together, 95.8% of the high risk VISCI inmates were detected by the SIRAS (see **Table 2**), whereas only 34.8% and 6.1% of the SIRAS high risk inmates were detected by the VISCI as red and yellow (see ESM 1).

**Table 2:** Distribution and Concordance of VISCI and SIRAS risk classification in the total sample.

		VISCI						Total	
		Green		Yellow		Red			
		n	%	n	%	n	%	n	%
SIRAS	Low	214	84.6%	8	66.7%	1	4.2%	223	77.2%
	High	39	15.4%	4	33.3%	23	95.8%	66	22.8%
	Total	253	100.0%	12	100.0%	24	100.0%	289	100.0%

VISCI, Viennese Instrument for Suicidality in Correctional Institutions; SIRAS, Scale for Initial Risk Assessment for Suicide. There are three risk categories in VISCI (green, low, red) and two in SIRAS (low, high).

**Table 3** summarizes the results for the surrogate markers for the total sample and for the risk classified inmates according to VISCI and SIRAS. Approximately 40% of all inmates were ordered to be placed on observation and night watch. The proportion was higher among the SIRAS high-risk inmates (each p <.001). According to VISCI, the proportion of these measures was also higher among yellow and red light inmates than among green light inmates, but this difference was not significant.

**Table 3:** Ordered measures in the total sample and in VISCI and SIRAS risk categories.

	Total sample (n = 289)	VISCI			SIRAS			
		Green (n = 253)	Yellow (n = 12)	Red (n = 24)	χ²(2)	Low (n = 223)	High (n = 66)	χ²(1)
Observation	40.5% (117)	38.7% (98)	41.7% (5)	58.3% (14)	3.05	31.8% (71)	69.7% (46)	30.29***
Night-time checks	40.8% (118)	38.7% (98)	41.7% (5)	62.5% (15)	5.13	32.3% (72)	69.7% (46)	29.50***
Consultation	15.2% (44)	11.5% (29)	33.3% (4)	45.8% (11)	23.24***	12.6% (28)	24.2% (16)	5.39*
Medication	20.4% (59)	16.5% (42)	41.7% (5)	50.0% (12)	18.53***	15.2% (34)	37.9% (25)	16.06***
Prison psychiatry	0.3% (1)	0.4% (1)	0% (0)	0% (0)	0.14	0.4% (1)	0% (0)	0.30

VISCI, Viennese Instrument for Suicidality in Correctional Institutions; SIRAS, Scale for Initial Risk Assessment for Suicide. *p <.05, ***p <.001. Significant values indicate that a measure was ordered more frequently in the risk group(s) than in the corresponding non-risk group.

One in six inmates (15.2%) had at least one follow-up visit with a psychiatrist or psychologist. The proportion was significantly higher among VISCI red (45.8%) and yellow light inmates (33.3%, p <.001), and also significant (but lower) among SIRAS high-risk inmates (24.2%, p <.01). A similar pattern emerged with regard to medication: one in five inmates received a medication prescription (20.4%), with the proportion significantly higher among VISCI red (50.0%) and yellow light inmates (41.7%, p <.001) and also among SIRAS high-risk inmates (37.9%, p <.001) compared to the corresponding low-risk inmates. Finally, only one inmate was transferred to the prison psychiatric unit and was not identified as high risk by either instrument.

## 4 Discussion

Effective suicide prevention in correctional settings requires the early identification of high-risk individuals, the provision of appropriate interventions, and the development of team-based interventions among staff. Although their use is the subject of critical debate, suicide screening instruments are generally recommended for use in the prison system (13). This study examined two suicide screening instruments (SIRAS and VISCI) in a sample of males inmates in a Berlin prison that were recently recommended by reviews (19, 25).

A key finding of this study is the magnitude of the difference in the classification of at-risk inmates. According to SIRAS, more than twice as many inmates would have been immediately referred to a psychiatrist or psychologist than according to VISCI (66 vs. 24 high-risk inmates). Please note that although the 12 yellow VISCI inmates indicate an increased risk, low-threshold measures (i.e. no single cell and work) are sufficient according to the manual (22). Even if these are included, SIRAS identifies almost twice as many inmates as high risk (23%) compared to the VISCI (12.5%). Comparative values from unselected samples are hardly available to assess the plausibility of the values. Only Dezsö et al. (26) can be mentioned here, in which 14% were identified as high risk using SIRAS, which is roughly consistent with the value here. No empirical data from independent studies are available for the VISCI. As no deaths by suicide were observed in the present study, no information can be provided on the diagnostic accuracy of the instruments. However, it can be assumed, that SIRAS is more sensitive but less specific to suicide risk. In other words, it can be assumed that SIRAS “misses” fewer people at risk. At the same time, this means that significantly more staff are needed for psychiatric or psychological consultation. In this context, Perry et al. (20) point out adverse effects of false-positive classifications (e.g., unnecessary investigations and treatments).

In terms of concordance between the instruments, the results show that all but one of the VISCI red lights were also identified as high risk by SIRAS. Conversely, only 35% of the high-risk inmates identified by SIRAS were also identified by VISCI. The concordance was largely due to acute suicidality: of the 23 inmates consistently classified as high risk, suicidal ideation or behavior was documented in 20 inmates. This leads to a second important finding of this study. Both instruments have some similarities, but more importantly, they have differences. They both capture prior incarceration, prior suicide attempts, and acute suicidal ideation, with the latter two being the most significant risk factors (8). Conversely, it became clear that the instruments reflect different risk areas: SIRAS relies more on substance use and sociodemographic characteristics (age, homelessness), while VISCI focuses on criminological (offense type) and psychiatric characteristics (contact with psychiatry and diagnosis), but also on sociodemographic characteristics (marital status, occupation). Of note, all the characteristics show individual robust associations with deaths by suicide in prison (8). At the same time, the results show that the instruments differ depending on the developmental sample and context and may miss relevant risk factors (especially the presence of a psychiatric disorder or a drug abuse history).

Finally, the results on the associations with the surrogate markers show that both instruments - at least partially - identify those inmates who received more monitoring and psychiatric or psychological support. As mentioned earlier no screening instrument was used at the time of the study and the screening results of this study were not reported back to the institution. On the one hand, this shows that mental health provision already seems to work to some extent (especially observation and consultation), albeit possibly for “other” reasons than suicidal tendencies (observation and night watch were ordered especially due to withdrawal symptoms). On the other hand, they can also be interpreted to mean that preventive mental health measures could be even more targeted. Suicide screening in addition to clinical assessments could help here, as the study by Deszö and Opitz-Welke (26) shows.

Based on these results and the literature, we would like to summarize some points that may be relevant when selecting an appropriate instrument in practice. First, predictive validity is similar (19, 25). However, independent and prospective studies are not available. In addition, it should be noted that the prediction of death by suicide by risk factors is not satisfactory, regardless of the context (16). Second, the instruments differ in the selection and composition of risk factors. For example, the SIRAS does not ask about psychiatric history and the VISCI does not ask about substance abuse. This can lead to very different risk classifications. An “independent” modification of the instruments could lead to an impairment of validity. In particular, the cut-offs could no longer be used. The only recommendation that can be made here is that these risk factors should be taken into account in the (clinical) assessment of acute suicidality. Other promising instruments could also be considered (27). Third, the SIRAS was developed in a pre-trial setting and, strictly speaking, can only be validly used in this setting. The VISCI, on the other hand, was developed in a mixed sample and explicitly takes custody status into account in the assessment. In addition, the VISCI has an additional level, the yellow category, with clear recommendations for action (i.e., no single cell and no work). Fourth, the greater differentiation of the VISCI can also be a disadvantage in practice: the VISCI contains more items that require more specific knowledge and is much more demanding to score. Depending on the custody status, different risk factors must be considered with different weightings. This is very cumbersome without technical support. In SIRAS, the assessment is much simpler. Although both tools are designed to be used by prison officers, the VISCI is definitely more sophisticated. It should be noted that even the use of SIRAS was described as burdensome by prison officers (26). In summary, it is not possible to make a clear recommendation for an instrument due to the methodological problems. Given the differences in practical application and “prison reality”, we would probably go with the SIRAS as the simpler tool. More important than the instrument seems to us to be the sensitization of staff to the mental health and suicidality of inmates. If the tools lead to inmates talking about this sensitive and often taboo subject, and not just at the beginning of their imprisonment, then much will have been achieved.

This study has several limitations that need to be considered. First, the file-based assessment may have biased the results, as some risk factors may have been present but not documented. This is why, for example, the VISCI specifies that the instrument be administered in the form of an interview. In addition, the assessment was completed by only one person. Although the study physician was well trained and most of the items can be answered relatively unambiguously (even on file basis), interrater reliability should be investigated in future studies. Second, death by suicide is a very rare event and fortunately none was detected in the present sample. Therefore, significant information about the diagnostic validity of the instruments could not be determined. However, it is precisely this information from independent studies that would be highly relevant in practice. Instead, and thirdly, surrogate markers were examined that could be confounded with the screening instruments due to the retrospective design (e.g., suicidal ideation was only known in a psychological interview and was not the reason for the consultation). Fourthly, the generalizability of the results is limited, as only men were examined. This is important because there are gender-specific differences (28). In addition, the characteristics of the facility with mixed pretrial and sentenced inmates, the regional characteristics of a metropolitan area, and German legislation may limit generalizability.

Suicide prevention in prison is a complex task in which suicide screening tools can only be a small component. However, the instruments only fulfill their purpose if they reliably enable the few available specialists to focus even more specifically on “at-risk” individuals. At the same time, the question of what the preventive function of screening instruments actually is needs to be asked. It can hardly be an accurate prediction of deaths by suicide with risk factors (16). It seems more likely that the actual function is to sensitize staff through their use and, above all, to actively raise the issue of suicidal ideation that might otherwise not be communicated. This requires further independent and methodologically high-quality studies. The present study could show that two instruments known mainly in German-speaking countries lead to very different assessments due to the risk factors taken into account. In practice, this should be a consideration in the selection of an instrument.

## Data availability statement

The raw data supporting the conclusions of this article will be made available upon reasonable request from the corresponding author.

## Ethics statement

Ethical approval was not required for the studies involving humans because the research was anonymized on a file basis without consequences for the sample. The studies were conducted in accordance with the local legislation and institutional requirements. Written informed consent for participation was not required from the participants or the participants’ legal guardians/next of kin in accordance with the national legislation and institutional requirements.

## Author contributions

JH: Data curation, Formal analysis, Methodology, Writing – original draft, Writing – review & editing. DC: Writing – review & editing. AO-W: Writing – review & editing.

## Footnotes

1 It is noteworthy that observation and night watch were most frequently ordered together (97.4%). The most common documented reason was withdrawal symptoms due to previous substance use as indicated by a positive drug/alcohol screening (92.3%), followed by mental health problems (7.7.%).
