# Supplementary material for: Comparison of two suicide screening instruments for identifying high-risk individuals in prison
Source: Front Psychiatry. 2024 Jun 27;15:1362928. doi: 10.3389/fpsyt.2024.1362928 (PMC11237454; doi:10.3389/fpsyt.2024.1362928)
Supplement: Supplementary file 1 [file Table_1.docx]

**Electronic supplementary material 1:**

Full table of distribution and concordance of VISCI and SIRAS risk classifications in the total sample.

|  |  |  | VISCI |  |  |
| --- | --- | --- | --- | --- | --- |
|  |  | Green | Yellow | Red | Total |
| SIRAS | Low | 214 | 8 | 1 | 223 |
|  | % of SIRAS | 96.0% | 3.6% | 0.4% | 100.0% |
|  | % of VISCI | 84.6% | 66.7% | 4.2% | 77.2% |
|  | High | 39 | 4 | 23 | 66 |
|  | % of SIRAS | 59.1% | 6.1% | 34.8% | 100.0% |
|  | % of VISCI | 15.4% | 33.3% | 95.8% | 22.8% |
|  | Total | 253 | 12 | 24 | 289 |
|  | % of SIRAS | 87.5% | 4.2% | 8.3% | 100.0% |
|  | % of VISCI | 100.0% | 100.0% | 100.0% | 100.0% |

*Note.* VISCI = Viennese Instrument for Suicidality in Correctional Institutions; SIRAS = Scale for Initial Risk Assessment for Suicide. There are three risk categories in VISCI (green, low, red) and two in SIRAS (low, high).
